# Improving uptake of pediatric vaccines through religious conferences and mobile vaccine clinics in Aceh, Indonesia (TABRIE): study protocol for a stepped wedge cluster randomized controlled trial

**DOI:** 10.1186/s13063-025-09170-5

**Published:** 2025-11-21

**Authors:** Rahul Ladhania, Ichsan Ichsan, Antonios M. Koumpias, Amanda Yufika, Rosaria Indah, Tita Menawati Liansyah, Abram L. Wagner, Harapan Harapan

**Affiliations:** 1 Department of Health Management and Policy, School of Public Health, University of Michigan, Ann Arbor, MI 48109 USA; 2 Department of Biostatistics, School of Public Health, University of Michigan, Ann Arbor, MI 48109 USA; 3 Center for Global Health Equity, Ann Arbor, MI 48109 USA; 4 Department of Family Medicine, School of Medicine, Universitas Syiah Kuala, Banda Aceh, 23111 Indonesia; 5 Medical Research Unit, School of Medicine, Universitas Syiah Kuala, Banda Aceh, 23111 Indonesia; 6 Department of Microbiology, School of Medicine, Universitas Syiah Kuala, Banda Aceh, 23111 Indonesia; 7 Tsunami & Disaster Mitigation Research Center (TDMRC), Universitas Syiah Kuala, Banda Aceh, 23111 Indonesia; 8 Department of Economics, University of Michigan, Dearborn, MI 48128 USA; 9 Medical Education Unit, School of Medicine, Universitas Syiah Kuala, Banda Aceh, 23111 Indonesia; 10 Department of Pediatrics, School of Medicine, Universitas Syiah Kuala, Banda Aceh, 23111 Indonesia; 11 Department of Epidemiology, School of Public Health, University of Michigan, Ann Arbor, MI 48109 USA; 12 Tropical Disease Centre, Universitas Syiah Kuala, Banda Aceh, 23111 Indonesia

**Keywords:** Routine pediatric vaccination, Vaccine hesitancy, Public health intervention, Mobile vaccine clinics, Religious leader conferences, Community health workers, Religious leaders, Stepped wedge cluster randomized controlled trial, Low- and middle-income countries, Indonesia

## Abstract

**Background:**

Despite advancements in child immunization, inadequate immunization rates in low- and middle-income countries persist due to inadequate health infrastructure, challenges in vaccine supply and distribution, insufficient healthcare provider training, and low levels of community trust in vaccines. Aceh, a religiously conservative province in Indonesia, has low pediatric vaccination coverage and exemplifies the need for innovative vaccine delivery models. Evidence suggests interventions should target both logistical barriers (e.g., distance or clinic wait times) and societal factors, including misinformation, that contribute towards vaccine hesitancy.

**Methods:**

The trial “TABRIE” will measure the impact of two strategies on children’s vaccination rates and parental attitudes towards vaccines in Banda Aceh and Aceh Besar, Indonesia, compared to current outreach strategies. The two strategies being tested are (a) an informational conference with religious leaders who work in specific clinics and (b) a mobile vaccine clinic staffed with community health workers conducting a variety of outreach events. We will execute a stepped wedge cluster randomized design with baseline measures and a cross-sectional sampling structure. Twelve districts (*Kecamatan*) will be randomized into one of the two strategies. In year 1, three districts from each strategy will implement the intervention, with the other three districts implementing the strategy in the second year. We will conduct cross-sectional surveys in September 2023 (baseline), September 2024 (year 1), and September 2025 (year 2). The primary outcome is the proportion of fully vaccinated children aged 1–5 years for bacillus Calmette-Guérin (BCG), diphtheria-tetanus-pertussis (DTP), polio, and measles. Secondary outcomes include the proportion of children aged 1–5 years with at least one dose of DTP and measles vaccines, the proportion of vaccine-hesitant parents, social norms surrounding vaccination, parental trust in community health workers to administer vaccines, the proportion of parents experiencing distance barriers to vaccination, the proportion of parents reporting that their religious leader encourages vaccination, and the proportion of parents receiving vaccination information from their religious leader.

**Discussion:**

This study will conduct a stepped wedge cluster randomized trial to separately estimate the effects of religious conferences and mobile vaccine clinics on pediatric vaccination rates and parental attitudes towards vaccination. It will offer a novel paradigm in vaccination delivery by inserting vaccination from clinics into social spaces that provide alternative, community-centered policy solutions to vaccine hesitancy.

**Trial registration:**

ClinicalTrials.gov NCT06160999. Registered on December 14, 2023.

## Administrative information

**Table:** 

Title {1}	A stepped wedge cluster randomized controlled trial for improving uptake of pediatric vaccines through religious conferences and mobile vaccine clinics in Aceh, Indonesia (TABRIE)
Trial registration {2a and 2b}	NCT06160999 [US Clinical Trials Register; registered on 14 December 2023; https://clinicaltrials.gov/study/NCT06160999]
Protocol version {3}	Protocol version 1.4, dated 6 November 2023
Funding {4}	This project is supported by the Center for Global Health Equity at the University of Michigan through the Global Vaccine Equity Initiative
Author details {5a}	University of Michigan, Ann Arbor, USA Universitas Syiah Kuala, Banda Aceh, Indonesia
Name and contact information for the trial sponsor {5b}	Center for Global Health Equity North Campus Research Complex 2800 Plymouth Road Building 100 Ann Arbor, MI, 48105, USA globalhealthequity@umich.edu
Role of sponsor {5c}	The study sponsor and funder had no role in study design; collection, management, analysis, or interpretation of data; writing of the report; or the decision to submit the report for publication

## Introduction

### Background and rationale {6a}

Despite significant advances in global childhood vaccination, immunization rates in many parts of the world remain insufficient. Vaccine disparities are particularly evident in low- and middle-income countries (LMICs), where coverage against diphtheria, pertussis, and tetanus—a key indicator of access to routine vaccination services—falls below 50% in many regions [1]. Coverage gaps have been further widened by the COVID-19 pandemic [2]. Barriers to vaccine distribution and utilization in LMICs include inadequate health facilities and local public health infrastructure, unreliable vaccine supply, storage, and transport capabilities, and lack of health care provider and/or proxy availability and training [3]. Behavioral and social drivers of vaccination are similarly important and include perceived disease risk, vaccine confidence, and social norms [4].

Although public approval of childhood vaccines is generally high in LMICs, there may be regional variations in acceptance and uptake that reveal inequities in vaccine access and/or confidence. According to the SAGE (Strategic Advisory Group of Experts on Immunization) Working Group on Vaccine Hesitancy, this lack of confidence may be manifested as vaccine hesitancy, defined as delay in acceptance or refusal of vaccines despite the availability of vaccination services [5]. Vaccine hesitancy is complex and content-specific and may be attributable to low levels of trust in vaccines and/or delivery systems; low perceived risk of vaccine-preventable disease; or barriers to accessibility, affordability, and availability, among other issues [6]. While health workers remain trusted sources of vaccine information and key influencers in immunization decision-making, they may face significant time constraints and resource limitations that impair their ability to administer vaccines and/or provide needed counseling. A major challenge, therefore, is the shortage of individuals who are culturally competent and equipped to administer vaccines and provide counseling. Ensuring that these individuals are well-trained and closely connected to the local communities they serve is critical.

Our study seeks to create a paradigm shift in how the public views and utilizes vaccination services. Currently, community health centers remain the default setting for vaccination, and clinicians the default administrators. However, the general population may have difficulty accessing these clinics or trusting traditional vaccination providers, particularly if they are members of marginalized communities that have experienced medical discrimination. Our study applies a two-pronged approach to address issues of trust and ease of access among the general population by (a) mobilizing religious communities to discuss vaccines (to counter reported lack of information about vaccines among unvaccinated families) and (b) training more community health workers in vaccination and in physically delivering vaccines through a mobile vaccine clinic (MVC) concept to facilitate ease in accessing vaccines. By mobilizing these individuals in the community settings where people live, work, worship, and learn, we aim to expand vaccine information and services. Through community engagement, we believe we can promote a participatory process in multiple aspects of the immunization program, a process that has been piloted in domestic settings [7].

More specifically, we plan to work with local health leaders in a low vaccination community in Aceh, Indonesia, to identify social institutions that are part of children and families’ daily lives; these could include houses of worship, schools, or community centers. We will fund a mobile vaccine delivery unit to go to these locations to physically bring vaccines to the people and to link them with existing immunization clinic infrastructure. We will also work towards changing the culture of child health and vaccination through substantial discussions and conversations with multiple levels of religious leaders at conferences. Overall, we hope to develop a simple and scalable training and recruitment program to equip community health workers to administer vaccines and religious leaders to provide culturally appropriate and specific counseling—all within familiar, comfortable social spaces. More than a one-time vaccination campaign, we hope to create a sustainable proxy network that can be deployed repeatedly, at intervals consistent with recommended vaccine schedules.

Case studies provide support for this approach. For example, a Pakistan provincial government deployed 13,000 women-led vaccination teams to local communities to provide COVID-19 immunization counseling, administration, and data entry [8]. In addition to traveling to people’s homes, these workers also offered vaccines in local temples, communal squares, and other public spaces. Another example would be the distribution of hepatitis B vaccines by trained community leaders at gay pride events [9]. MVCs have been positively viewed in a variety of settings—including COVID-19 clinics in Boston, universities in the USA, and in emergency settings in places like Afghanistan, Haiti, and Palestine [10–12]. However, there is limited information about their routine use in LMIC settings. Our study builds on these previous models by inserting vaccination into diverse social spaces, simultaneously “liberating” vaccine administration from clinics and increasing convenient access to vaccines. Our model has the potential to reach children and families that are currently excluded from receiving vaccines or information from more formal, clinic-based vaccination services.

This protocol describes the TABRIE study which aims to evaluate the impact of two vaccine uptake strategies on children vaccination rates and parental attitudes towards vaccines in Banda Aceh and Aceh Besar, two study locations in the province of Aceh, Indonesia.

### Objectives {7}

The primary objective of the study is to examine the impact of the two interventions—MVC and religious conferences—on full vaccination coverage at the district level. The secondary objective is to examine the effect of these interventions on uptakes of specific vaccines, measures of vaccine hesitancy, and other barriers to vaccination uptake. While the study is not powered for a direct head-to-head comparison between MVC and religious conferences, we will perform exploratory analyses comparing the two interventions and interpret the results cautiously. Primary objective measure will be the proportion of children 1–5 years of age fully vaccinated (receipt of bacillus Calmette-Guérin, diphtheria-tetanus-pertussis, polio, and measles vaccines).

Hypothesis for primary objective: We expect that the intervention arms will have greater vaccination coverage than the non-intervention arms.

Secondary objective measures: (a) proportion of children 1–5 years of age with one dose of diphtheria-tetanus-pertussis vaccine; (b) proportion of children 1–5 years of age with a measles vaccine; (c) proportion of parents vaccine hesitant; (d) proportion of parents stating a social norm towards vaccination; (e) proportion of parents with trust in a community health worker to administer vaccines; (f) proportion of parents stating that distance is a barrier to vaccination; (g) proportion of parents stating that their religious leader wants them to be vaccinated; and (h) proportion of parents receiving vaccination information from their religious leader.

Hypothesis for secondary objective: Our secondary objectives are exploratory and we do not present pre-existing hypotheses.

### Trial design {8}

The 2-year study is conducted in a stepped wedge cluster randomized design [13]. Our clustered unit of analysis is a district, or *Kecamatan*, in Indonesia. Because we have two interventions, we are running two concurrent trials. Each intervention will involve 6 districts, for 12 districts involved total. Each district typically has one or two *Puskesmas* (*Pusat Kesehatan Masyarakat*) or community health clinics—the location of most vaccinations. Each district also is composed of several villages.

Our study location is Banda Aceh, the provincial capital of Aceh, and Aceh Besar, the surrounding suburban area. There are a total of 41 districts in Banda Aceh and Aceh Besar. We randomly sampled 18 of these. We subsequently performed a cluster analysis, where we clustered these 18 districts into 2 groups based on the following district-level characteristics: (a) population density; (b) *Puskesmas* building conditions—variables indicating the condition of *Puskesmas* buildings, such as good, lightly damaged, moderately damaged, and heavily damaged; (c) healthcare personnel—number of general practitioners, midwives, and community health workers in each district; (d) auxiliary *Puskesmas* conditions including good, lightly damaged, moderately damaged, and heavily damaged; (e) educational institutions—number of *Poskestren* (Islamic boarding schools); (f) healthcare facilities—number of beds and maternity care beds available; and (g) internet network availability—status of internet networks, including good, not smooth, and none.

**Fig. 1:**
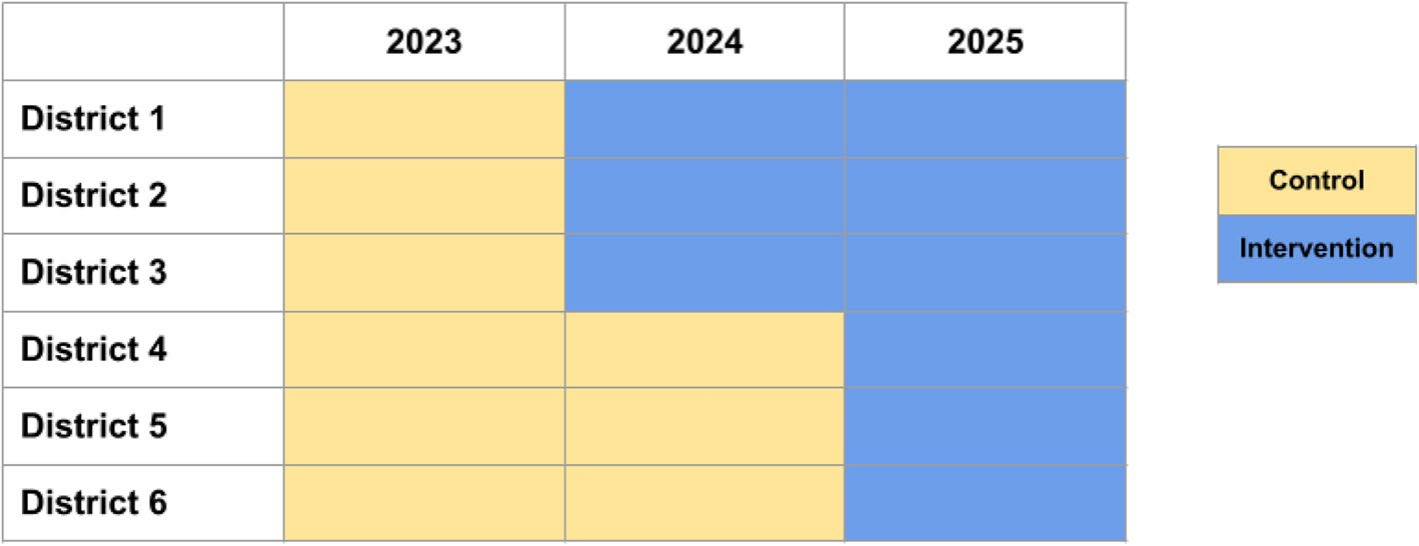
Modified stepped wedge design of the TABRIE study. 1.Notes: We are running two concurrent interventions - Mobile Vaccine Clinic (MVC) and Religious Conference. Each intervention involves 6 districts. 2023 was the baseline year with no intervention assignment

The first group had 12 districts, and the second six districts. For the first 12 districts, we additionally removed six districts whose population center was close to the population center of another district to avoid spillovers. In this way, we ended with six districts in each group. We then randomly assigned the two groups to either the MVC strategy or the religious conference strategy. Thus, the six districts from the first group were assigned to the MVC strategy, and the six districts in the second group were assigned to the religious conference strategy (Table 1). For each group of 6, 3 districts were from Banda Aceh, and 3 from Aceh Besar. Within each group, we then randomized three districts to begin the intervention in year 1 and three in year 2 using a computer-generated allocation. District authorities were informed only after randomization. Although the strategies were implemented simultaneously, analytically, these are treated as two separate trials. The stepped wedge design and the district schedule for the two interventions are depicted in Fig. 1 and Table 1.

**Table 1:** Sites of the two interventions, TABRIE, Aceh, Indonesia, 2023–2025

Strategy	Year(s) of intervention (randomized)	Location	District
Mobile vaccine clinic (MVC)	1 and 2	Banda Aceh	Baiturrahman
MVC	1 and 2	Banda Aceh	Jaya Baru
MVC	1 and 2	Aceh Besar	Mesjid Raya
MVC	2	Banda Aceh	Kuta Raja
MVC	2	Aceh Besar	Peukan Bada
MVC	2	Aceh Besar	Krueng Barona Jaya
Religious conference	1 and 2	Aceh Besar	Kuta Malaka
Religious conference	1 and 2	Aceh Besar	Darussalam
Religious conference	1 and 2	Banda Aceh	Meuraxa
Religious conference	2	Banda Aceh	Kuta Alam
Religious conference	2	Banda Aceh	Lueng Bata
Religious conference	2	Aceh Besar	Montasik

Year of intervention start was randomized within each intervention arm (3 districts began in year 1 and 3 in year 2)

## Methods: participants, interventions, and outcomes

### Study setting {9}

Aceh is a religiously conservative region in Indonesia and has been granted more autonomy by the national government. From a recent paper we wrote using 2017 data, pediatric measles vaccination coverage was 54% in Aceh, the lowest of any province in Indonesia, indicating a need for more innovative vaccine delivery models [14]. A recent study of social media use in Indonesia found that vaccine misinformation clustered around concerns about halal certification, industrial conspiracies, and concerns about side effects [15].

### Eligibility criteria {10}

To be eligible for the study; the participant should: (a) adults 18 or older; (b) proficient in Bahasa Indonesia; (c) plan to live in neighborhood for next year; and (d) parent of child <5. Eligibility criteria apply to the surveyed household population (children 1–5 years of age and their parents) which is the basis of the primary outcome.

### Who will take informed consent? {26a}

Study staff will be trained in ethical considerations and study procedures and will be responsible for obtaining informed consent. For participant recruitment, study staff will randomly select households from the birth registry at the Puskesmas that include children meeting the study criteria. The selected households will then be contacted by telephone, and if they agree to participate, the interview will be conducted either in their homes or at designated locations. Study staff will explain the contents of the form, ensuring participants understand the purpose of the study, their rights, and the voluntary nature of participation. Before starting the questionnaire, staff will ask, “Is this a good place and time to talk?” If privacy concerns arise, the interviewer will either identify a more private location or arrange to return later to complete the interview.

All informed consent procedures will be conducted by the trained study staff. We will document written informed consent. Participants will receive a copy of the informed consent form for their records.

### Additional consent provisions for collection and use of participant data and biological specimens {26b}

N/A.

## Interventions

### Explanation for the choice of comparators {6b}

The control groups receive standard outreach from healthcare workers, i.e., *Puskesmas* staff conduct regularly scheduled outreach activities in each of the villages in the control districts. This is the comparator of choice since the aim of the study is to assess if the two strategies lead to increased vaccination coverage compared to current outreach activities.

### Intervention description {11a}

The study comprises two distinct interventions. The first intervention is a religious conference that will bring together subdistrict-level imams, other religious leaders, and a variety of community health workers, including those not traditionally trained to administer vaccines. The conference will feature both joint sessions with all participants and breakout sessions tailored by profession. The topics covered during the conference will be developed collaboratively with the religious leaders, with a primary focus on the importance of infant health and vaccination. After the religious conference, we will also follow up with some religious leaders at later points in the year.

The second intervention is a mobile vaccine clinic (MVC), where a community health worker will travel to different locations within the test districts. These locations will be chosen in collaboration with the local health department and based on the research team’s understanding of the area. Targeted sites will include spaces frequented by families with young children, such as schools, mosques, and sports fields. The purpose of the mobile clinic is not only to deliver vaccines but also to personalize vaccine delivery by introducing a human connection—represented by the community health worker—outside of a clinical setting. The community health worker will be equipped to administer vaccines and address any questions or concerns individuals may have.

### Relevant concomitant care permitted or prohibited during the trial {11d}

N/A.

### Provisions for post-trial care {30}

N/A.

### Outcomes {12}

The primary outcome is full vaccination, which is ascertained through records from vaccination cards or clinic records. Secondary outcomes are also listed in Table 2, along with the original measure in the survey instrument, and how it is re-coded (as applicable).

**Table 2:** Primary and secondary outcome measures

**#**	**Description**	**Measurement**	**Recode**
Primary outcome			
1	Full vaccination	Records from vaccination cards or clinic records 6 months since the start of the intervention: 1 dose of BCG; 3 doses of DTP; 3 doses of polio vaccine; 1 dose of measles-containing vaccine	
Secondary outcomes			
2a	DTP dose 1 vaccination	Records from vaccination cards or clinic records	
2b	Measles dose 1 vaccination	Records from vaccination cards or clinic records	
2c	Vaccine hesitancy	Overall, how hesitant about childhood shots would you consider yourself to be ● Not at all hesitant (1) ● Not that hesitant (2) ● Somewhat hesitant (3) ● Very hesitant (4) ● Unsure (5) ● Refused (6)	Dichotomized (3 + 4 is hesitant, other responses and missing responses count as non-hesitant)
2d	Social norms of vaccination	Do you think most parents you know vaccinate their children? ● Yes (1) ● No (2) ● Don’t know (3)	Dichotomized (1 is yes, other responses and missing responses count as no)
2e	Trust in community health workers	How much would you trust your child being vaccinated by a community health worker in a mobile vaccine clinic? ● Strongly distrust (1) ● Distrust (2) ● Neither trust or distrust (3) ● Trust (4) ● Strongly trust (5)	Dichotomized (4 + 5 is trust, other responses and missing responses count as do not trust)
2f	Distance barrier	What barriers have you encountered in getting your children vaccinated? (Select all that apply) ● [Other options provided] ● The distance from my residence to the vaccination site is too far (6)	Dichotomized (selecting 6 is yes, other responses and missing responses count as no)
2g	Vaccination norms from religious leaders	Do you think your religious leaders want you to vaccinate your children? ● Yes (1) ● No (2) ● Don’t know (3)	Dichotomized (1 is yes, other responses and missing responses count as no)
2h	Information from religious leaders	How and where did you hear information about the vaccination program for your children? (Select all that apply) ● [Other options provided] ● From religious leaders (6)	Dichotomized (selecting 6 is yes, other responses and missing responses count as no)

### Participant timeline {13}

**Fig. 2:**
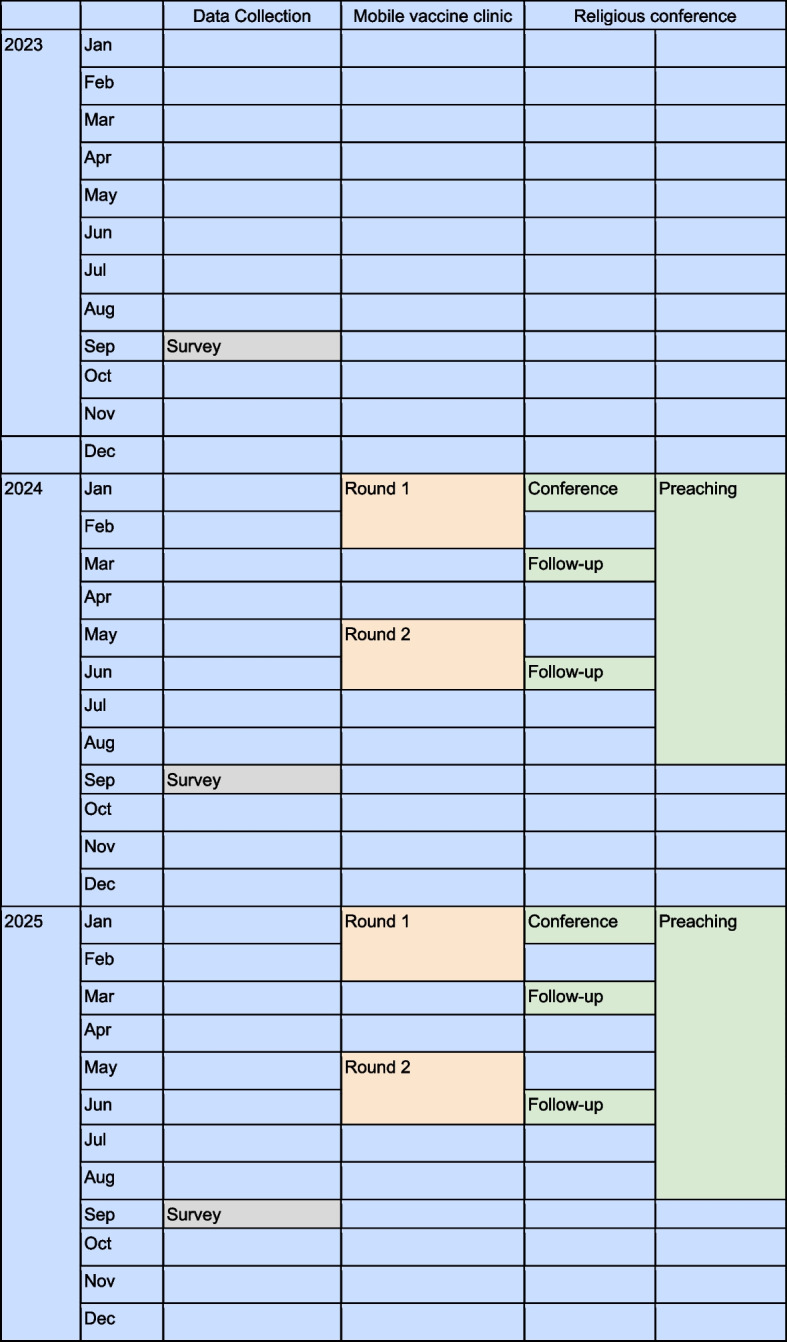
Timeline of intervention strategies in a stepped wedge cluster randomized control trial, TABRIE, Aceh, Indonesia, 2023–2025

Cross-sectional surveys will be conducted September of each year from 2023 to 2025. Baseline year is 2023, year 1 of the interventions is 2024, and year 2 is 2025. In each year, a religious conference will be held in January. We expect that participants will disseminate vaccine promotions to their communities after this date. We will also follow up with participants from the conference in March and June. For the MVCs, community health workers will conduct outreach events in two rounds. Round 1 will be in January and February, and round 2 will be in May and June (Fig. 2).

### Sample size {14}

For this cluster randomized control trial, we specified a stepped wedge design with baseline measures and a cross-sectional sampling structure. The primary outcome is the net difference between arms in repeated cross-sectional samples. We used the following parameters for sample size calculation: (a) type I error rate: 0.05; (b) power: 0.80; (c) expected vaccination proportion in controls: 49.5% (based on the 2022 survey); and (d) intraclass correlation coefficient (ICC): 0.0816 (based on the 2022 survey).

To detect a 13 percentage point increase in vaccination coverage (62.5% in the intervention arm vs. 49.5% in the control arm), we determined that 100 individuals per cluster are required. With a stepped wedge design involving three clusters in the first year and three in the second year, this sample size provides adequate power to detect the specified effect size. The sample size calculation assumed an exchangeable (time-invariant) intracluster correlation; no temporal decay was assumed.

### Recruitment {15}

We are conducting a series of cross-sectional surveys as part of this trial. For each district, we will obtain a household listing with a child aged 1–5 years from the *Puskesmas* catchment area and will randomly select the households. Enumerators will contact the selected households by telephone, and if they agree to participate, the interview will be conducted either in their homes or at designated locations. If a household refuses or is ineligible, the next randomly drawn household will be contacted and approached. Survey enumerators do not disclose intervention allocation or timing to sampled households. Communities might be informed of the upcoming MVC or religious conferences through the routine mobilization processes that are part of the individual interventions.

## Assignment of interventions: allocation

### Sequence generation {16a}

N/A.

### Concealment mechanism {16b}

N/A.

### Implementation {16c}

In this stepped wedge design, participants’ intervention is determined by where they live. Participants are enrolled into the study by a team of trained enumerators.

## Assignment of interventions: blinding

### Who will be blinded {17a}

N/A.

### Procedure for unblinding if needed {17b}

N/A.

## Data collection and management

### Plans for assessment and collection of outcomes {18a}

Protocol and data collection instruments can be found here: 10.6084/m9.figshare.25991875.

### Plans to promote participant retention and complete follow-up {18b}

N/A.

### Data management {19}

As data is collected and entered into a secure database, the study team will regularly tabulate results for the primary and secondary objectives to monitor data quality issues. Although we do not anticipate adverse events being observable in the data, regular quality checks will ensure completeness, accuracy, and consistency.

Compliance with regulatory documentation and study data integrity will be maintained through an internal quality assurance process implemented by the study team.

Confidentiality will be safeguarded throughout the trial. Informed consent forms, enrollment forms, and questionnaires will be stored in separate secure locations. After the questionnaires are input into an electronic database, the physical copies will be destroyed to further protect participant information. Informed consent forms will be retained for the duration specified by the institutional review boards (IRBs).

The data will initially be stored on a secure, HIPAA-compliant online platform—such as Dropbox—that is supported by the University of Michigan. While data collection is ongoing and some personally identifiable information may temporarily reside in the database, access to the folder will be password-protected and restricted to authorized study team members only.

### Confidentiality {27}

Reproducibility and replicability are critical to advancing scientific knowledge and ensuring transparency in research. To this end, we plan to upload the final dataset, along with detailed statistical analysis code, to a public repository such as Figshare. Prior to publication, all personally identifiable information will be removed from the dataset to protect participant confidentiality. Additionally, geographic locations will be anonymized by replacing specific identifiers with coded numbers, ensuring that individuals cannot be located or identified.

### Plans for collection, laboratory evaluation, and storage of biological specimens for genetic or molecular analysis in this trial/future use {33}

Not applicable.

## Statistical methods

### Statistical methods for primary and secondary outcomes {20a}

We will report descriptive statistics, including demographic characteristics by cluster and by intervention arm.

The primary outcome is a binary variable: full vaccination (Table 2). For the main analyses, we will include one randomly selected child aged 1–5 years from each family. All models will adjust for the following pre-specified covariates: (a) respondent characteristics such as age, gender, education (less than high school, high school, more than high school), and ethnicity (Acehnese vs. non-Acehnese) and (b) child characteristics such as age (1-, 2-, 3-, 4-year-old), gender, and birth order.

### Primary outcome analysis: mixed-effects logistic regression

To evaluate the primary outcome, we will use a generalized linear mixed model (GLMM) model with a logit link, including period-fixed effects and a cluster-level random intercept to account for intracluster correlation. The outcome of full vaccination will be specified as the dependent variable, with experimental condition as a dummy variable to estimate the treatment effect.

- We will include data from all years and all clusters, adjusting for time and covariates

$$\begin{aligned} logit(P({Y}_{ijk}=1))={\beta}_{0} &\,+{\beta}_{1}\cdot Treatment_{jk}+\gamma_{1}1\{k=1\}\\ &\,+\gamma_{2}1\{k=2\} + X_{ijk}^T\beta_2+{u}_{j} \end{aligned}$$

${\beta }_{1}$: treatment effect (log-odds)

${\gamma }_{k}$: time trends independent of the treatment, where $\textrm{k} \in \{1,2\}$

### Secondary analysis: time-by-treatment interaction

- To test whether the intervention effect varies over time, we will include interaction terms between treatment and period. As no clusters are treated in Year 0 (baseline) and all are treated in Year 2, we parametrize the model with interaction terms and period fixed effects.

$$\begin{aligned}&logit(P(Y_{ijk}=1))\\ \quad \quad \quad \quad \quad & =\beta_0+\gamma_1 1\{k=1\}+\gamma_2 1\{k=2\} \\\quad \quad \quad \quad \quad &+ \beta_{11}(Treatment_{jk}\times 1\{k=1\}) \\\quad \quad \quad \quad \quad &+ \beta_{12}(Treatment_{jk}\times 1\{k=2\})\\\quad \quad \quad \quad \quad &+X_{ijk}^T\beta_2+u_j\end{aligned}$$

$\beta_{11}$ and $\beta_{12}$ are treatment effects (log-odds) in Year 1 and Year 2 respectively. We will conduct additional analyses for which we have no directional hypothesis or preliminary data to guide sample size considerations. Analyses of our secondary outcomes listed in Table 2 (e.g., specific vaccines, vaccination attitudes) will use analogous GLMMs appropriate to the outcome type. We will also conduct mediation analyses, to ascertain if the secondary outcomes are on the mediation pathway between the experimental conditions and the primary outcome. We will also do subgroup analysis based on individual-level characteristics (e.g., age, gender, baseline covariates). All data will be analyzed in R or SAS.

### Interim analyses {21b}

We will assess the preliminary effect of the intervention while preserving trial integrity.

### Descriptive analysis

- Summarize outcomes (proportion with binary outcome) by cluster for baseline year and year 1.
- Compare intervention vs. control clusters descriptively at the end of year 1.
- Summarize key baseline individual- and cluster-level covariates by treatment group (for treated clusters) and control group (for untreated clusters at year 1).

### Mixed-effects logistic regression for preliminary treatment effects

- Fit a binary outcome model with a cluster-level random effect (to account for intracluster correlation)

where:

- *i*: individual, j: cluster, w *k∈ {0,1,2}: year*
- $Treatmen{t}_{jk}$: indicator variable for treatment status in cluster j in year k
- $$NA$$
- ${X}_{ijk}$: covariates listed above
- ${u}_{j}$: cluster-level random intercept

$$logit(P({Y}_{ijk}=1))={\beta }_{0}+{\beta }_{1}\cdot Treatmen{t}_{jk}+\gamma_{1}1\{k=1\} +X_{ijk}^T{\beta }_{2}+{u}_{j}$$

Here ${\beta }_{1}$ is the log-odds ratio for the preliminary treatment effect.

### Methods for additional analyses (e.g., subgroup analyses) {20b}

Use machine learning to identify subgroups or predictors of differential treatment effects:

- Use causal forests to estimate treatment effect heterogeneity [16].
- Outcomes:
  - Identify groups for which the treatment effect is stronger or weaker.
  - Generate hypotheses about interaction terms for inclusion in traditional models.

Given our study’s limited power, subgroup analyses will be exploratory, estimated via treatment and covariate interaction terms at the individual level. Subgroup analyses will use “honest” estimation approaches (e.g., sample splitting to separate model training from estimation) from the causal machine learning literature to avoid over-interpretation, and results will be interpreted cautiously using established frameworks such as the ICEMAN tool to assess credibility [17].

### Methods in analysis to handle protocol non-adherence and any statistical methods to handle missing data {20c}

Our main analysis will be based on participants who have provided all required information for the primary outcome.

### Plans to give access to the full protocol, participant-level data, and statistical code {31c}

The full protocol is publicly available: 10.6084/m9.figshare.25991875. Statistical code will be uploaded with each manuscript. The de-identified participant-level dataset will also be made publicly available.

## Oversight and monitoring

### Composition of the coordinating center and trial steering committee {5d}

The research teams in Indonesia (led by Dr. Harapan) and the USA (led by Dr. Wagner) provide day to day support for the trial. The team in Indonesia meets weekly, and the two sides meet every 2 weeks during intensive data collection and trial implementation periods, otherwise monthly.

### Composition of the data monitoring committee, its role and reporting structure {21a}

Because we are examining a group-level outcome, a data monitoring committee is not applicable.

### Adverse event reporting and harms {22}

This study is a cluster randomized control trial, with districts receiving different vaccine-related interventions, including a MVC and religious conference. While individuals within those districts may benefit from these interventions, the individual participants in the study may or may not experience direct benefits.

The primary risk associated with the study is the potential loss of confidentiality regarding participants’ children and their vaccination information. However, this risk is considered low, as the data collected during the study is not highly granular. Additionally, we have established a robust data protocol to minimize this risk. We will store all questionnaires in a password-protected folder on Dropbox, a HIPAA-compliant platform supported by the University of Michigan.

We have structures in place to manage and address potential negative reactions. Although we do not anticipate adverse reactions, as the interventions—MVCs and religious conferences—are extensions of well-established public health programs, the research team remains prepared to respond. As a team of researchers in the USA and Indonesia, we will regularly be in contact to discuss this project. Indonesian researchers have already engaged community members, including religious leaders and public health workers, to promote transparency and build trust. As part of a community-engaged approach, the Indonesian team will conduct quarterly follow-ups with stakeholders to assess the intervention’s effectiveness, identify any concerns, and address potential negative reactions. If we observe adverse trends, such as reduced vaccination uptake or increased vaccine hesitancy linked to the interventions, we will pause the implementation to investigate further. We will identify specific subpopulations showing negative reactions and organize focus groups to explore the underlying causes and inform modifications to the interventions.

These strategies will ensure that risks are actively mitigated, and any harms are promptly addressed to safeguard the well-being of participants and the broader community.

### Frequency and plans for auditing trial conduct {23}

We do not currently have plans to audit the trial.

### Plans for communicating important protocol amendments to relevant parties (e.g., trial participants, ethical committees) {25}

Not applicable (the study is repeated cross-sectional in design).

### Dissemination plans {31a}

We note no publication restrictions. Findings from the study will be publicized on the funder (Center for Global Health Equity) website, in peer-reviewed manuscripts, and in technical reports tailored to an Indonesian audience.

## Discussion

The TABRIE trial seeks to address persistent challenges in improving pediatric vaccination rates by targeting both logistical barriers and societal factors. By combining mobile vaccine delivery with tailored engagement through religious leaders, the trial offers an innovative approach to integrating vaccination services into community settings. The stepped wedge cluster randomized design is particularly suited to this context, allowing for the equitable and phased rollout of interventions while providing robust internal validity for assessing their effects on vaccination rates and parental attitudes.

The trial builds on global evidence demonstrating the importance of integrating vaccination into familiar social spaces, such as religious institutions, schools, and community centers, to counteract barriers like vaccine hesitancy and access limitations. Past successes of MVCs and culturally tailored counseling—seen in Pakistan, Boston, and elsewhere—support the potential of this model. TABRIE extends these approaches by engaging religious leaders and community health workers in Aceh, Indonesia, to provide culturally sensitive counseling and increase trust in vaccination services. This dual strategy addresses the intertwined barriers of logistical inaccessibility and misinformation that disproportionately affect underserved populations.

Operational challenges emerged during year 2, particularly in the Jaya Baru district of Banda Aceh, where some healthcare workers refused to cooperate with the study team. While other villages within Jaya Baru permitted the intervention, the district could not be replaced due to its prior exposure to treatment and the structure of the trial design. To account for this, we will conduct sensitivity analyses using a “leave-one-out” approach to evaluate the robustness of our findings with and without data from Jaya Baru. Although these analyses may have limited power, they will provide valuable insight into the potential impact of this issue.

This trial contributes to a growing body of work emphasizing the importance of tailoring vaccination delivery strategies to local contexts. By combining evidence-based logistical interventions with culturally appropriate outreach, TABRIE aims to provide practical and scalable solutions for increasing vaccine uptake. Its findings will offer actionable insights for policymakers and practitioners seeking to address vaccine hesitancy and improve immunization coverage in similar low-resource settings.

## Trial status

Current protocol version: 1.4, updated 6 November 2023.

Recruitment began 1 September 2023. We expect recruitment to finish 31 December 2025.

## Data Availability

A de-identified final dataset will be made publicly available.

## Abbreviations

BCG: Bacillus Calmette-Guérin
COVID-19: Coronavirus disease 2019
DTP: Diphtheria-tetanus-pertussis
HIPAA: Health Insurance Portability and Accountability Act
IRB: Institutional Review Board
LMICs: Low- and middle-income countries
MVC: Mobile vaccine clinic
NCT: National Clinical Trial
Puskesmas: *Pusat* *Kesehatan* *Masyarakat* (Community health clinic)
SAGE: Strategic Advisory Group of Experts on Immunization
SPIRIT: Standard Protocol Items: Recommendations for Interventional Trials
TABRIE: An Acehnese word meaning “We deliver”, referring to our study: Improving Uptake of Pediatric Vaccines Through Religious Conferences and Mobile Vaccine Clinics in Aceh, Indonesia
